# Evaluation of the Back College for nursing staff

**DOI:** 10.1186/s12995-014-0032-7

**Published:** 2014-09-20

**Authors:** Peter Koch, Aki Pietsch, Melanie Harling, Susanne Behl-Schön, Albert Nienhaus

**Affiliations:** 1 Centre of Excellence for Epidemiology and Health Services Research for Healthcare Professionals (CVcare), University Medical Centre Hamburg-Eppendorf, Martinistraße 52, Hamburg, 20246, Germany; 2 Rehabilitation Centre City Hamburg, Lange Mühren 1, Hamburg, 20095, Germany; 3 Health Protection Division (FBG), Institution for Statutory Accident Insurance and Prevention in the Health and Welfare Services (BGW), Pappelallee 33, Hamburg, 22089, Germany

**Keywords:** Back school, Nursing, Evaluation

## Abstract

**Background:**

Work-related musculoskeletal pain- particularly back pain - is an important individual and socioeconomic problem. The Back College for the insurance holders of the Institution for Statutory Accident Insurance and Prevention in the Health and Welfare Services (BGW) is based on a multimodal concept and has been evaluated with respect to pain relief and continuing in the nursing profession.

**Methods:**

In a retrospective cohort study, the participants in the Back College from 2009 to 2011 were surveyed in writing. Besides demographic data, the survey covered information on qualification, length of employment, institution, employment status, periods of inability to work, applicability of working techniques and continuation in the profession. Back pain was recorded at three time points - T1 (before the Back College), T2 (directly after the Back College) and T3 (at the time of the survey). Pain changes were submitted to tests for paired samples. Multivariate logistic analysis was applied to determine potential factors influencing unfavourable changes in pain or leaving nursing due to back pain.

**Results:**

The survey covered 1,282 insurance holders, with a response rate of 80%. Statistically significant reductions in pain were found for the whole group and for all subgroups. For persons who predominantly worked in old people’s homes and who did not take part in refresher services, an increased odds ratio was found for unfavourable changes in pain (OR: 1.9 or 1.4, respectively). Persons with a qualification in geriatric nursing or in intensive care/OP/anaesthesia had an increased risk of leaving nursing due to back pain (OR: 2.5 in each case). An increased risk of leaving was also found for persons who did not take part in workplace support (OR: 2.9).

**Conclusion:**

Within the context of the study design, the multimodal concept of the Back College is clearly related to relief of back pain. The Back College appears to be less successful for geriatric nurses and persons with qualifications in intensive care/OP/anaesthesia. Further studies are needed to ascertain why some participants experience less relief in stress from the working techniques they have learnt.

## 1 Background

Mobilisation, transfer and supporting patients in everyday activities expose nursing staff to a high degree of physical stress. For this reason, musculoskeletal diseases are of considerable importance in this occupational group [1],[2]. In comparison to other occupational groups, professional nurses are at increased risk of back pain [3] and have a six-fold higher prevalence of damage to the back [4]. Problems with the lumbar spine are thought to be the main reason that nursing staff leave their profession [5]. Both awkward postures during patient transfer and psychosocial factors increase the risk of back pain in nursing staff [6]. In order to decrease the stress to which nurses are exposed, a variety of intervention programs have been developed to prevent back pain [7]. Multimodal intervention programs for nursing staff exhibit greater efficacy than individual interventions [8]-[10].

### 1.1 The Back College - a measure for secondary individual prevention

To reduce back pain and its risk factors, intensive physical training and cognitive behaviour modification elements were combined in a multimodal intervention. Thus, the Back College is a multimodal measure for secondary individual prevention. It is made available to insurance holders of the Institution for Statutory Accident Insurance and Prevention in the Health and Welfare Services (BGW) who already suffer from back pain due to degenerative changes in the vertebral discs of the lumbar spine. This measure serves to prevent an imminent occupational disease and premature discontinuation of the profession. The Back College has been performed since 1994 by Rehabilitation Centre City Hamburg.

The 3-week program of the Back College [11] includes *physical therapy, physiotherapy* and *sport medical training therapy*. This is intended to strengthen the muscles and enhance basic physical stability and somaesthesia, together with the automatisation of movement patterns. In addition, the Back College focuses on *training* in *occupational-specific practises*. After making allowance for technical and organisational factors, participants are taught how to perform different types of patient transfer while sparing their backs. The main component is training in somaesthesia, equilibrium and coordination when displacing weight when shoving and pulling loads during the working process. This intervention is complemented by *psychological health training* when dealing with pain and stress. In this way, the participants learn to understand the multifactorial conditions under which pain arises and is processed, so that they recognise how their own thoughts and behaviour influence the pain symptoms. They are to learn how to deal with their pain and stressful situations in an active and independent manner. Additional information is provided in a *lecture from a physician* on the biomechanics of the spinal column, *nutritional advice*, *training in medical devices and aids*, as well as a *lecture from a BGW representative* on conditions related to the vertebral discs of the lumbar spine as occupational diseases (BK 2108).

In addition, about 12 weeks after the Back College, the participants are offered *workplace support*, in order to check the implementation of the new techniques at the workplace. The employees are motivated to continue to use behaviour to spare their backs and are supported by a working environment which spares the back.

Another follow-up module is the 5-day *refresher course*. This is offered to participants between 12 and 18 months after the Back College. The material in the Back College is then revised and problems are discussed that cropped up during daily work after the Back College.

#### 1.1.1 Questions to be answered

In this study, the following central questions were formulated:

1. How does the intensity of the participants’ back pain change after the Back College in comparison to previously?

2. Which factors related to the participants influence unfavourable changes in pain?

3. Which factors related to the participants influence their leaving the nursing profession due to back pain?

## 2 Methods

### 2.1 Study design and data collection

The Back College was evaluated in 2012 with a retrospective cohort study. Thus, in September 2012, all BGW insurance holders were contacted for whom suspected BK 2108 was reported during the period 2009–2011 and who had taken part in the Back College. For persons who had moved to an unknown address, a search was performed using the Municipal Register for Residents.

The study was tuned with the data protection commissioner of the BGW. Insurance holders were contacted with an anonym questionnaire. The clarification writing included the information that in case of a completed sent back questionnaire, the subject declared its consent in participation. Because this observational study was performed as an anonym survey no approval by an ethics committee was collected.

Aside from *demographic information*, the questionnaire contained information on *qualification, length of employment, institution, employment status, periods of inability to work* and *the reasons for these, applicability and transmission of the working techniques learnt, use of other BGW services and satisfaction with the intervention setting. Back pain* was recorded on a 10-point pain scale at three time points - T1 (before the Back College), T2 (directly after the Back College) and T3 (at the time of the survey). The data of T1 and T2 were recorded retrospectively. To evaluate work ability, two questions from the work ability index questionnaire [12] were integrated in the questionnaire.

#### 2.1.1 Statistical evaluation

To evaluate the differences in the pain distribution at times T1 and T3, tests for paired samples (Wilcoxon signed rang test) were performed for the whole group and for the subgroups. In order to study possible factors influencing changes in pain, the difference in pain at time points T1 and T3 was calculated and dichotomised over the mean. The mean (*X*^−^ = 1.47) was rounded up, leading to a limit in the scale at a value of 2, i.e. all values of ≥ 2 were defined as favourable and all values < 2 as unfavourable changes in pain. A lack of pain relief was defined as an outcome and odds ratios were calculated using a multivariate logistic regression procedure. The Hosmer & Lemeshow [13] stepwise backwards’ method was used, in which variables with p > 0.1 were successively excluded. The following variables were used for model building: *age, gender, qualification, period in nursing, institution, number of symptoms, applicability of working techniques, relief from working techniques, employment, year of the Back College, other BGW services and pain at time point T1.*

The third question was about the factors influencing discontinuing work as a nurse. To address this, persons who discontinued working as a nurse due to back pain were continued with persons still employed in nursing. Thus, this evaluation excluded persons who left nursing for other reasons. Here too the regression procedures were performed as described above and the same variables were included - aside from employment. The refresher course was excluded from this analysis too, as the data do not say when the refresher course took place. It was therefore unclear whether professional drop-outs had the responsibility to take part in the refresher course.

The evaluation was performed with the statistics package SPSS Version 21.

## 3 Results

### 3.1 Description of the cohort

Questionnaires were sent to 1,742; a total of 1,394 questionnaires were returned (response rate: 80%). As a result of the enquiries to the Municipal Register for Residents, 140 new addresses were identified and 62 additional persons. After selecting participants who exclusively worked as nurses, there remained a total cohort of 1,282 persons. Table 1 describes the characteristics of the cohort.

**Table 1:** Description of the cohort and pain relief

			**Frequency**	**Percent**	Pain development T1-T3*		
**Total group** N = 1394	Not employed in nursing		112	**8%**			
	Employed in nursing		1282	**92%**			
					**Median T1**	**Median T3**	**Test (p)**
**Employed in nursing**			1282	**100%**	6	4	< 0.001
**Year of Back College**	2009		425	33.2%	6	4	< 0.001
	2010		388	30.3%	6	4	< 0.001
	2011		469	36.6%	6	4	< 0.001
	Missing		0	0%			
**Gender**	Female		1141	89%	6	4	< 0.001
	Male		140	10.9%	5	4	< 0.001
	Missing		1	0.1%			
**Age at Survey**	20-29		25	2%	6	2.5	< 0.001
	30-39		95	7.4%	6	4	< 0.001
	40-49		406	31.7%	6	4	< 0.001
	50-59		601	46.9%	6	4	< 0.001
	≥ 60		145	11.3%	6	5	< 0.001
	Missing		10	0.8%			
**Year in nursing**	0-9		203	15.8%	6	4	< 0.001
	10-19		443	34.6%	6	4	< 0.001
	20-29		395	30.8%	6	4	< 0.001
	≥ 30		233	18.2%	6	4	< 0.001
	Missing		8	0.6%			
**Qualification**	Nursing		646	50.4%	6	4	< 0.001
	Nursing assistant		233	18.2%	7	5	< 0.001
	Paediatric nursing/Obstetrics		18	1.4%	6	5	0.015
	Geriatrics		289	22.5%	6	5	< 0.001
	Nursing management		9	0.7%	5	3	0.039
	Intensive care/OP/anaesthetics		87	6.8%	6	4	< 0.001
	Missing		0	0%			
**Institution**	Outpatient nursing		181	14.1%	6	4	< 0.001
	Hospital		630	49.1%	6	4	< 0.001
	Old people’s home		425	33.2%	6	5	< 0.001
	Other		46	3.6%	6	4	< 0.001
	Missing		0	0%			
**Employment**	Yes		1100	85.8%	6	4	< 0.001
	No		179	14%	7	5	< 0.001
	Missing		3	0.2%			
**BGW services**	Refresher course		514	40.1%	6	3	< 0.001
	Personal advice		197	15.4%	6	4	< 0.001
	Workplace support		376	29.3%	6	4	< 0.001
	Outpatient rehabilitation measures		40	3.1%	7	5	< 0.001
	Aids for patient transfer		74	5.8%	6	4	< 0.001
	Other		38	3%	7	4	0.002
	No services		38	3%	6	4.5	0.007
	Missing		4	0.3%			
**Applicability of working techniques in occupation**	Yes		1044	81.4%	6	4	< 0.001
	No		216	16.8%	7	5	< 0.001
	Missing		22	1.7%			
	If yes: reduction in stress to the lumbar spine with working techniques	Yes	911	87.3%	6	4	< 0.001
		No	108	10.3%	7	6	0.046
		Missing	25	2.4%			

*Missing values for pain development T1-T3: N = 79.

The participants for the three years were roughly evenly distributed within the cohort (Table 1). There was a clear majority of women (89%). The frequency in the age groups increased continuously, reaching a peak in the 50–59 age group (47.2%). About 35% of the participants stated that they had worked in nursing for 10–19 years. Only about 18% had worked for more than 30 years. As regards qualifications, half the participants were trained as nurses, followed by training in geriatric nursing (22.5%). Half of the participants stated that they had worked most of the time in hospital. 33.2% of the participants worked mostly in old people’s homes. A smaller proportion (other) reported that they had predominantly worked in homes for the handicapped. As regards employment, 86% reported that they worked in nursing or in another area.

### 3.2 Other BGW services used

About 40% of the cohort stated that they had taken the 1-week refresher course. About 15% had taken advantage of personal advice from a BGW representative. A workplace support was used by about 29%. Services that were much more rarely used included outpatient rehabilitation measures (3.1%), aids for patient transfer (5.8%) and other measures (e.g. back consultation, reimbursement for physiotherapy) (3%). 3% of the participants stated that they did not draw on any offers of the BGW to reduce back stress.

### 3.3 Applicability of the working techniques

A very high proportion of the participants (81%) considered that the working techniques learnt in the Back College could be applied at work. Moreover, about 87% of these persons considered that they felt that these working techniques had relieved stress on the lumbar spine.

### 3.4 Satisfaction with the components of the Back College

**Figure 1:**
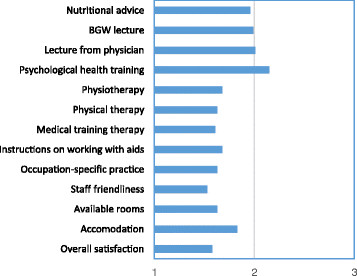
Rating of the Back College components with the school marking system (1 = very good, 6 = inadequate).

Figure 1 shows the rating of the individual components and the overall satisfaction with the Back College. Overall satisfaction was given an intermediate rating of 1.58 in the school marking system. Staff friendliness was given the best rating (1.53). All other individual components lay in the range between 1.61 (medical training therapy) and 2.15 (psychological health training). In summary, the participants were highly satisfied with the Back College in general, and with its individual components.

### 3.5 Periods of inability to work and working ability

At the time of the survey, 195 (15.2%) of the participants reported that they were currently unable to work (Table 2). This was caused by problems with the lumbar spine (52.3%), the thoracic spine (16.4%) or the cervical spine (21.0%). More than half of the persons unable to work (65.6%) reported that this was caused by other health problems.

**Table 2:** Current inability to work, with reasons

**Current inability to work**	**Frequency**	**Percent**
Yes	195	15.2%
No	1070	83.5%
Missing	17	1.3%
**If yes: reasons for inability (multiple answers allowed):**		
Lumbar spine symptoms	102	52.3%
Thoracic spine symptoms	32	16.4%
Cervical spine symptoms	41	21.0%
Other health problems	128	65.6%
Missing	8	4.1%

The following answers were made to the question whether inability to work during the past 12 months was exclusively due to lumbar spine symptoms. A total of 426 (33.2%) persons stated that they had been unable to work during the previous 12 months due to lumbar spine symptoms (Table 3). For the different institutions, this proportion ranged between 30.3% (outpatient nursing) and 34.8% (other). About 28% of the group reported inability to work (sick leave) for up to 2 weeks. 26% reported periods of 2 to 4 weeks and about 10% reported inability to work for between 4 and 6 weeks. About 35% reported long term inability to work - for more than 6 weeks. As regards the institution, the highest percentage of inability to work for more than 3 months was found for the staff of old people’s homes, 26.2% of whom reported inability to work for between 3 and 12 months.

**Table 3:** Periods of inability to work from lumbar spine symptoms / 12 months

**Inability to work during the last 12 months**	**Outpatient nursing N = 181**	**Hospital N = 630**	**Old people’s home N = 425**	**Other N = 46**	**Total N = 1,282**
< 2 weeks	40.0% (22)	29.0% (62)	19.1% (27)	43.8% (7)	27.7% (118)
2-4 weeks	16.4% (9)	29.0% (62)	25.5% (36)	31.3% (5)	26.3% (112)
4-6 weeks	5.5% (3)	8.9% (19)	15.6% (22)	6.3% (1)	10.6% (45)
6 weeks-3 months	16.4% (9)	14.0% (30)	13.5% (19)	0.0% (0)	13.6% (58)
3-12 months	21.8% (12)	19.2% (41)	26.2% (37)	18.8% (3)	21.8% (93)
**Total**	**30.3% (55)**	**34% (214)**	**33.2% (141)**	**34.8% (16)**	**33.2% (426)**

Table 4 shows the subjective evaluation of current working ability in comparison to the best level ever attained. According to this, about 16% considered that their current working ability was slight. About one third (30.4%) described their current working ability as moderate and about half as high. In this respect, there was essentially no difference between the different institutions (table not shown). There was a similar distribution in answers to the question as to what extent the current work will be possible during the next two years: just under 13% thought it was improbable, 35.5% were not sure and almost half the cohort (49%) were certain that their state of health would permit them to carry out their current work during the next two years.

**Table 4:** Current and predicted subjective working ability

**Current working ability**	**Frequency**	**Percent**
Slight (0–3)	209	16.3%
Moderate (4–6)	390	30.4%
High (7–10)	641	50%
Missing	42	3.3%
**Performing current work in 2 years**		
Improbable	161	12.6%
Uncertain	455	35.5%
Fairly certain	635	49.5%
Missing	31	2.4%

### 3.6 Pain development

**Figure 2:**
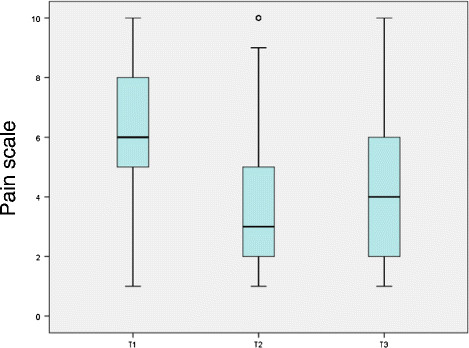
Distribution of pain at time points T1, T2, T3.

**Figure 3:**
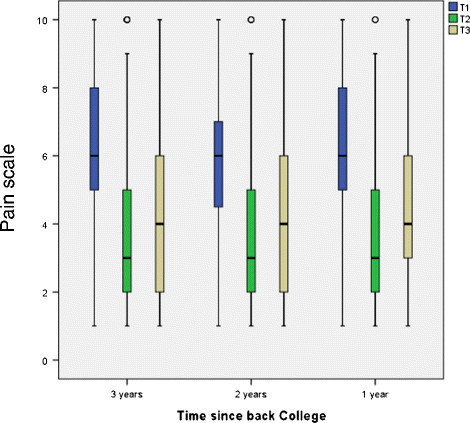
Pain development relative to time since Back College.

Pain was compared at T2 - directly after the Back College (median = 3) - with T1 - before the Back College (median = 6). There was a marked decrease in pain for the whole group (Figure 2). At the time of the survey (T3), pain had slightly risen again. The median was then 4, which is still better than the value at T1. Relative to the time since the Back College, identical medians were found for all three years at T1, T2 and T3 (Figure 3). Thus, there was no time-dependent increase in the pain level at T3 (the time of the survey) for 2009 and 2010.

Table 1 gives the medians of the pain distributions at T1 and T3 for the total group and the subgroups. The total cohort exhibits a statistically significant pain reduction. For the three years of the Back College - 2009–2011 - the pain reductions shown in Figure 3 were statistically significant. The pain reductions were also statistically significant in all the listed subgroups: gender, age groups, employment period, qualification, institution, employment, BGW services and applicability of the working techniques. The greatest pain reduction was in the 20 to 29 age group, where the median decreased from 6 to 2.5. The second greatest reduction was in persons who took part in the refresher course (median T1: 6, median T3: 3).

Table 5 shows the pain categories (slight/moderate/intense) at time points T1 and T3 for the whole cohort. The proportion of participants with slight pain before the Back College (24.2%) was doubled at the time of the survey (51.2%). The proportion of persons with moderate pain fell from 48.4% to 30.4% and the proportion with intense pain from 24.2% to 13.3%. This pain reduction is statistically significant in the paired sample test (p < 0.001).

**Table 5:** Pain in categories at time points T1 and T3

**Pain**	**Before the Back College T1**		**At time of survey T3**		**Test T1-T3 (p)**
	**Frequency**	**Percent**	**Frequency**	**Percent**	
**Slight pain (1–4)**	310	24.2%	657	51.2%	p < 0.001
**Moderate pain (5–7)**	620	48.4%	390	30.4%	
**Intense pain (8–10)**	210	24.2%	171	13.3%	
**Missing**	42	3.3%	64	5%	
**Total**	1,282	100%	1,282	100%	

The following is observed for the target variable pain relief (Table 6): about 45% of participants experienced pain relief of at least 20% (≥2 scale points). About half of the group (48.9%) had experienced no pain relief at the time of the survey. In this subgroup, there was a statistically significant increase in the median at T3 relative to T1 (median: 5 versus 6).

**Table 6:** Pain development T1-T3 in the outcome parameters

**Target variable**	**Frequency**	**Percent**	**Median T1**	**Median T3**	**Test (p)**
**Pain relief**	576	44.9%	7	3	<0.001
**No pain relief**	627	48.9%	5	6	<0.001
**Missing**	79	6.2%			
**Total**	1282	100%			
**Leaving nursing due to back pain**	132	11.5%	7	6	<0.001
**Currently employed in nursing**	949	82.6%	6	4	<0.001
**Missing**	68	5.9%			
**Total**	1149	100%			

**Figure 4:**
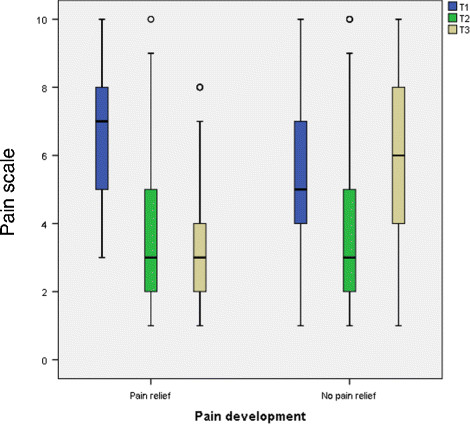
Pain development in the group variable pain relief.

For the group of persons who experienced pain relief, there was a clear trend over time (Figure 4). Between T1 and T2, the median decreased from 7 to 3, but then remained constant to T3, but with decreased scatter. In contrast, in the comparator group without pain relief, initial pain was lower (median: 5). At T2, the median decreased to 3, but at T3 increased to above the initial value (median 6).

The analysis for leaving nursing excluded persons who left nursing for other reasons (e.g. retirement, further training, other health reasons) (Table 6). At the time of the survey, 82.6% were working in nursing. 132 persons reported that they had left nursing due to back pain (11.5%). For both groups, there was a statistically significant reduction in pain intensity. For the group who left nursing, the decrease in the median (from 7 to 6) was less than for the comparator group.

### 3.7 Factors influencing unfavourable pain development

Multivariate analysis was performed for the factors influencing unfavourable pain development, defining *lack of pain relief* as the outcome. Table 7 shows the statistically relevant factors (p < 0.1), together with the final model of the logistic regression analysis. As expected, the risk for lack of pain relief increased with increasing age class. For persons who predominantly worked in old people’s homes, there was a statistically increased odds ratio of 1.9 (95%CI: 1.29-2.93) relative to persons who worked in outpatient nursing. Back College participants who did not take part in the refresher course exhibited an increased odds ratio of 1.4 (95%CI: 1.07-1.82) for lack of pain relief. For persons who stated that the working techniques they had learnt did not relieve stress on the spinal column, there was a 3.7-fold increased odds ratio for lack of pain relief (OR: 3.7 95%CI: 2.73-5.05).

**Table 7:** **Results of the log. Regression, dependent variable** ***pain relief*** **(N = 1,149)**

**Variable**		**Pain relief**		p*	**Final model OR (95% CI)**
		**No% (n)**	**Yes% (n)**		
**Age**	20-39	41.9%% (49)	58.1% (68)	**0.005**	1
	40-49	51% (198)	49% (190)		1.4 (0.85-2.17)
	50-59	52% (294)	48% (271)		1.5 (0.98-2.41)
	≥ 60	64.5 (80)	35.5% (44)		**2.2 (1.24-3.94)**
**Years in nursing**	0-9	42% (78)	58% (108)	**0.017**	-
	10-19	53% (218)	47% (196)		
	20-29	56% (211)	44% (165)		
	≥ 30	53% (116)	47% (103)		
**Qualification**	Nursing	48% (305)	52% (331)	**0.012**	
	Nursing assistant	54.5% (116)	45.5% (97)		
	Geriatric nursing	56.8% (150)	43.2% (114)		
	Intensive care/OP/anaesthetics	62.2% (56)	37.8% (34)		
**Institution**	Outpatient nursing	45% (77)	55% (94)	0.072	1
	Hospital	51.2% (308)	48.8% (294)		1.3 (0.88-1.90)
	Other	60.5% (26)	39.5% (17)		1.9 (0.86-4.12)
	Old people’s home	55.8% (216)	44.2% (171)		**1.9 (1.29-2.93)**
**Employment**	Yes	51% (526)	49% (510)	**0.028**	-
	No	60% (99)	40% (66)		
**Refresher course**	Yes	47.8% (223)	53.2% (254)	**0.003**	1
	No	55.5% (402)	44.5 (322)		**1.4 (1.07-1.82)**
**Applicability of working techniques**	Yes	49.1% (479)	50.9% (497)	**< 0.001**	-
	No	67.6% (140)	32.4% (67)		
**Relief of lumbar spine with working techniques**	Yes	45% (383)	55% (468)	**< 0.001**	1
	No	70.9% (219)	29.1% (90)		**3.7 (2.73-5.06)**
**Pain before the Back College**	Intense	39.5% (120)	60.5% (184)	**< 0.001**	1
	Moderate	47.9% (289)	52.1% (314)		**1.8 (1.30-2.45)**
	Slight	73.6% (218)	26.4% (78)		**7.2 (4.83-10.61)**
*χ^2^ (Pearson)				R^2^: 0.21	

### 3.8 Leaving nursing due to back pain

**Figure 5:**
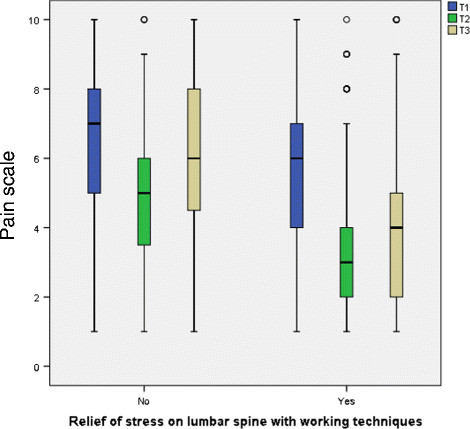
Pain development after relief of stress on lumbar spine with working techniques.

Table 8 shows the results of the logistic regression for leaving nursing due to back pain. In comparison to persons with training in hospital nursing, the odds ratio was statistically significantly increased for persons with a qualification in geriatric nursing - by a factor of 2.5 (OR: 2.5 95%CI: 1.53-4.21). The same increased odds ratio was found for persons with training in intensive care/OP/anaesthetics (OR: 2.5 95%CI: 1.22-5.52). 28.6% of the persons who reported that they felt no relief in the lumbar spine had left nursing. Relative to the persons who felt relief to the lumbar spine, this corresponded to an increased odds ratio by a factor of 5.3 (OR: 5.3 95%CI: 3.48-8.14). This group not only exhibited greater initial pain, but also had relatively high pain scores at T2 and T3 (Figure 5). For persons who did not use the service of workplace support from a BGW representative, there was a statistically significant increase odds ratio (OR: 2.9 95%CI: 1.55-5.31). The following trend was found for the variable *pain before the Back College:* the odds ratio for leaving nursing was increased by a statistically significant factor of 2.3 for persons with moderate pain and by a factor of 5.1 for persons with intense pain.

**Table 8:** **Results of the log. Regression, dependent variable** ***leaving nursing*** **(N = 1,065)**

***Variable***		***Leaving nursing***		p*	**Final model OR (95% CI)**
		***Yes***	***No***		
***Age***	*20-39*	*9.0%(9)*	*91%(91)*	**<0.001**	1
	*40-49*	*8.2%(31)*	*91.8%(345)*		0.82 (0.35-1.96)
	*50-59*	*11.2%(61)*	*88.8%(484)*		0.98 (0.43-2.24)
	*≥ 60*	*31.1%(37)*	*68.9%(82)*		**3.39 (1.38-8.31)**
***Qualification***	*Nursing*	*7.9%(48)*	*92.1%(557)*	**<0.001**	1
	*Nursing assistant*	*13.7%(28)*	*86.3%(176)*		1.2 (0.65-2.16)
	*Geriatric nursing*	*18.8%(47)*	*81.2%(203)*		**2.5 (1.53-4.21)**
	*Intensive care /OP/anaesthetics*	*16.7%(15)*	*83.3%(75)*		**2.5 (1.22-5.52)**
***Years in nursing***	*0-9*	*8.1%(14)*	*91.9%(159)*	**0.002**	-
	*10-19*	*16.5%(65)*	*83.5%(329)*		
	*20-29*	*8.5%(31)*	*91.5%(334)*		
	*≥ 30*	*13.3%(28)*	*86.7%(182)*		
***Institution***	*Outpatient nursing*	*17.5%(28)*	*82.5%(132)*	**0.026**	-
	*Hospital*	*9.7%(56)*	*90.3%(520)*		
	*Other*	*7.3%(3)*	*92.7%(38)*		
	*Old people’s home*	*13.7%(51)*	*86.3%(321)*		
***Workplace inspection***	*Yes*	*4.9%(17)*	*95.1%(329)*	**<0.001**	1
	*No*	*15.1%(121)*	*84.9%(681)*		**2.9 (1.55-5.31)**
***New aids***	*Yes*	*3%(2)*	*97%(65)*	**0.019**	-
	*No*	*12.6%(136)*	*87.4%(945)*		
***Number of sites of symptoms***	*One*	*9.9%(68)*	*90.1%(618)*	**0.013**	-
	*Two*	*14.9%(47)*	*85.1%(269)*		
	*Three*	*17.3%(23)*	*82.7%(110)*		
***Applicability of working techniques in occupation***	*Yes*	*7.6%(71)*	*92.4%(866)*	**<0.001**	-
	*No*	*31.9%(61)*	*68.1%(130)*		
***Reduction in stress on lumbar spine from working techniques***	*Yes*	*5.7%(47)*	*94.3%(771)*	**<0.001**	1
	*No*	*28.6%(82)*	*71.4%(205)*		**5.3 (3.48-8.14)**
***Adequate training in implementing working techniques***	*Yes*	*8.8%(56)*	*91.2%(580)*	**0.021**	-
	*No*	*13.2%(60)*	*86.8%(396)*		
***Time since Back College***	*1 year*	*9.9% (42)*	*90.1% (381)*	0.241	1
	*2 years*	*13.6% (49)*	*86.4% (311)*		1.6 (0.93-2.69)
	*3 years*	*12.8% (47)*	*87.2% (319)*		1.1 (0.65-1.88)
***Pain before Back College***	*Slight*	*4.3%(12)*	*95.7%(268)*	**<0.001**	1
	*Moderate*	*10.8%(60)*	*89.2%493)*		**2.3 (1.15-4.74)**
	*Intense*	*22.9%(64)*	*77.1%(216)*		**5.1 (2.45-10.52)**
χ^2^ (Pearson)				R^2^ : 0.31	

## 4 Discussion

The first question to ask is whether the Back College program is associated with favourable effects on pain development in the participants in the Back College for the years 2009–2011. This retrospective cohort study found statistically significant pain relief for the whole group and for the subgroups. Identical pain reductions were found for the three years, which indicates that the intervention is sustainable. The observed pain relief is also supported by the ease in applying the working techniques and the marked satisfaction with the Back College.

The factors which have an unfavourable effect on pain relief were also considered. It was found that persons who did not take part in the refresher course exhibited a statistically significant increased odds ratio of 40%. In comparison with persons working in outpatient nursing, persons who mostly worked in old people’s homes exhibited a statistically significant increased odds ratio of 90%. In addition, persons who reported no relief on the lumbar spine with the working techniques were at a statistically significant increased odds ratio of 3.7.

The factors that influence the participants to leave nursing were also considered. An increased odds ratio was found for persons who did not take part in workplace support (OR: 2.9). Increases in odds ratios were also observed for employees with a qualification in geriatric nursing (OR: 2.5) or in intensive care /OP/anaesthetics (OR: 2.5). There was also a statistically significant increased odds ratio for persons who reported no relief on the lumbar spine with the working techniques (OR: 5.3).

### 4.1 Limitations

As the response rate was 80%, the cohort was relatively well covered. As no responder analysis was performed, we are unable to state whether the missing group of persons differed in any way and whether this led to bias in the results.

The most suitable way to evaluate the efficacy of an intervention, such as the Back College, to relief back stress would be to use a study design such as a randomised controlled trial. However, it is difficult to recruit a suitable control group, as the BGW as accident insurer is obliged by the German Social Code to make every effort to reduce the risk that insurance holders suffer the occurrence, recurrence or deterioration of an occupational disease. This is the legal basis that all insurance holders have the right to the Back College - a secondary prevention program. It is therefore only possible to perform intraindividual and internal comparisons in subgroups. In contrast, the 2003 evaluation study of the Back College [14] exploited the unique opportunity that, at this time, the Back College was under development and was not accessible to all BGW insurance holders. In this context, it was possible to perform a non-randomised controlled evaluation study with group comparison.

Moreover, the retrospective design is susceptible to recall bias. It is possible that participants tended to report more pain for time point T1 and less pain for time point T3. Information on pain development might also be influenced by the authority of the BGW and perhaps by the wish to use other BGW services in future. Also, as in all interventional studies, bias due to social desirability cannot be ruled out in the participants’ response behaviour.

Other influences that might also have a positive effect on the perception of pain have not been assessed in this study. This refers to medical treatment e.g. usage of pain relievers and physiotherapy. Additionally the influence of personality in terms of cognitive and behavioural characteristics e.g. pain coping strategies were not assessed [15]. Also adjustment for medical conditions before the Back College was not performed.

Other known risk factors for back pain include physical, ergonomic and psychosocial factors [6],[16],[17] but were not considered in this study. As a result, the survey instrument was relatively brief and this might be one reason for the good response rate.

Risk estimates in this retrospective cohort study are slightly overestimated. Due to frequent events especially for pain development odds ratios overestimate the relative risk and have to be interpreted with caution.

### 4.2 Pain development

In the context of the study design, statistically significant pain reduction was found for the whole group and the subgroups. This is consistent with the 2003 evaluation study of the Back College [14]. This study too found pain relief in active participants at the time of the survey.

In addition, 81% of the participants considered that the working techniques they had learnt were easy to apply. 87% of participants felt relief to the lumbar spine. Similarly good application rates have been published [18],[19]. The identical pain relief for the three years also seem to indicate that the intervention is sustainable. High quality studies in related literature find similar results for the efficacy of multimodal interventions. In the review by Dawson et al. [10] a moderate level of evidence is found for multimodal interventions in preventing back pain. The findings of Hignett [8] also underline the results of this study: on the basis of 10 studies a moderate level of evidence was stated for the effectiveness of multimodal interventions. However, it is also possible that the increased odds ratio for employees in old people’s homes (OR:1.9) and the higher rates of long-term working inability due to symptoms in the lumbar spine may reflect the comparatively high stress of working in an old people’s home [20].

A study in Germany showed that aids for patient transfer are rarely provided or used in inpatient geriatric care [21]. In addition, greater functional impairment due to pain in the back of the neck and back has been found in European geriatric nurses than in hospital or outpatient nurses [22]. The stress from an unfavourable psychosocial situation may also be linked to back pain in geriatric nurses [22]. In contrast, an alternative explanation for the increased risk may be that the Back College is not optimally designed for geriatric nurses. An argument against this view is that the module *occupation-specific practice* deals with the specific areas in which the participants work [11].

The increased odds ratio for persons who did not participate in the refresher course (OR: 1.4) may indicate that the refresher course is an effective follow-up module of the Back College. An alternative explanation would be a healthy user bias [23], i.e. the group of refresher participants are generally more health conscious and have less pain for this reason. However, a decision of this sort may also have been made when deciding to participate in the Back College.

The increased odds ratio for persons who reported no relief on the lumbar spine with the working techniques might be explained as follows. For some reason, the working techniques were not learnt and consequently no pain relief was observed. On the other hand, these may be people with more severe damage to the lumbar spine. This idea is supported by both the higher initial pain in this subgroup, as well as the lesser pain reduction, which is only just statistically significant (median: from 7 to 6 vs. from 6 to 4). Thus there could perhaps be a group of participants suffering from relatively severe back pain and who therefore benefit more from the therapeutic arm of the Back College and only have suboptimal characteristics for the preventive arm.

### 4.3 Leaving nursing due to back pain

Because of the correlation between the characteristic qualification in geriatric nursing and the characteristic old people’s home, it is probable that in this case too the increased odds ratio in persons with a qualification in geriatric nursing (OR: 2.5) reflects the higher working stress in geriatric nursing. A study on new entrants analyses lower back pain as an independent risk factor for leaving geriatric nursing [24]. An analysis of data of the German statutory pension insurance assumes that geriatric nurses have a higher risk for a reduced work ability on the basis of musculoskeletal disorders than nurses working in a hospital [25]. The observed risk for leaving geriatric nursing complies with the results of a study investigating the nursing situation in Germany. Independent of the reason for leaving the profession it is shown that personnel in geriatric nursing have a shorter duration (8.4 years) in the profession than nursing personnel in hospitals (13.7 years) [26]. This premature drop-out of geriatric nurses is also confirmed in the NEXT study [27], showing that geriatric nurses are more likely to intend to leave their job than nurses working in the hospital. The increased risk for persons with intensive care /OP/anaesthetics training (OR: 2.5) presumably also reflect working conditions that predispose to leaving nursing due to back pain. It is a fact that operating theatre nurses have to stand for long periods and this is typical of the stress on the back when working in this area.

The increased odds ratio of leaving for persons who did not participate in workplace support (OR: 2.9) indicates that this is an effective follow-up module. However, the participation rate in this timely and setting-related measure is low (29.4%), so that only a small proportion of the nursing staff currently take part. As all participants in the Back College currently obtain a recommendation for workplace support, the low participation is probably due to the employee and/or the unit.

As with the analysis of pain development, persons who felt no relief in the lumbar spine exhibited an increased odds ratio for leaving nursing (OR: 5.3). This association too indicates a subgroup who leave nursing prematurely, presumably due to an advanced stage of pain.

## 5 Conclusions

This evaluation study shows that the Back College of Hamburg City Rehabilitation Centre is associated with relief in noticed back pain in nursing staff. The results of this study are in line with the existing results of studies investigating the efficacy of multimodal interventions for the prevention of back pain. In order to verify this result, a prospective study should be performed. It would also be desirable to investigate whether the 3-week intervention is helpful for persons with intense initial pain or serious damage to the back. It must be clarified to what extent the instruction on prevention is helpful for these people. Perhaps pure rehabilitation would be better for this group.

For geriatric nurses and nurses in intensive care /OP/anaesthesia, one possibility would be to perform on site risk assessment, in order to adapt the module *occupational-specific practice* to the actual working environment. It is possible that there are working conditions in these areas that have not yet been properly considered in this module.

## Abbreviations

BGW: Institution for Statutory Accident Insurance and Prevention in the Health and Welfare Services

BK 2108: Occupational disease related to the vertebral discs of the lumbar spine

CI: Confidence interval

OP: Operation theatre

OR: Odds ratio

T1: Before the Back College

T2: Directly after the Back College

T3: At the time of the survey

*X*^−^: Mean

## Competing interests

Peter Koch has no conflict of interests. Aki Pietsch is responsible for implementing the Back College at the Hamburg City Centre for Rehabilitation Medicine. Melanie Harling has no conflict of interests. Susanne Behl-Schön has no conflict of interests. Albert Nienhaus has no conflict of interests.

## Authors’ contributions

PK, performed the survey, carried out the statistical analyses and wrote the manuscript. AP gave insight to the processes and the curriculum of the Back College and was critically reading the manuscript. MH read the draft critically and gave substantial comments for the improvement of the first draft. SB-S supported the writing process in presenting the curriculum of the Back College. AN revised the manuscript critically for important intellectual content and gave final approval for the version to be published. All authors read and approved the final manuscript.

## References

[B1] Feyer AM Herbison P Williamson AM de Silva I Mandryk J Hendrie L Hely MC The role of physical and psychological factors in occupational low back pain: a prospective cohort study Occup Environ Med 2000 57 116 120 10.1136/oem.57.2.116 10711279 PMC1739913

[B2] Josephson M Lagerström M Hagberg M Wigaeus HE Musculoskeletal symptoms and job strain among nursing personnel: a study over a three year period Occup Environ Med 1997 54 681 685 10.1136/oem.54.9.681 9423583 PMC1128844

[B3] Hofmann F Stössel U Michaelis M Nübling M Siegel A Low back pain and lumbago-sciatica in nurses and a reference group of clerks: results of a comparative prevalence study in Germany Int Arch Occup Environ Health 2002 75 484 490 10.1007/s00420-002-0332-6 12172895

[B4] Cohen-Mansfield J Culpepper WJ Carter P Nursing staff back injuries: prevalence and cost in long term care facilities AAOHN J 1996 44 9 17 8694975

[B5] Nelson A Fragala G Menzel N Myths and facts about back injuries in nursing Am J Nurs 2003 103 32 40 10.1097/00000446-200302000-00021 12582336

[B6] Sherehiy B Karwowski W Marek T Relationship between risk factors and musculoskeletal disorders in the nursing profession: a systematic review Occup Ergon 2004 4 241 279

[B7] Hignett S Work-related back pain in nurses J Adv Nurs 1996 23 1238 1246 10.1046/j.1365-2648.1996.13423.x 8796474

[B8] Hignett S Intervention strategies to reduce musculoskeletal injuries associated with handling patients: a systematic review Occup Environ Med 2003 60 E6 10.1136/oem.60.9.e6 12937202 PMC1740617

[B9] Tullar JM Brewer S Amick BC III Irvin E Mahood Q Pompeii LA Wang A Van ED Gimeno D Evanoff B Occupational safety and health interventions to reduce musculoskeletal symptoms in the health care sector J Occup Rehabil 2010 20 199 219 10.1007/s10926-010-9231-y 20221676

[B10] Dawson AP McLennan SN Schiller SD Jull GA Hodges PW Stewart S Interventions to prevent back pain and back injury in nurses: a systematic review Occup Environ Med 2007 64 642 650 10.1136/oem.2006.030643 17522134 PMC2078392

[B11] http://www.bgw-online.de/SharedDocs/Downloads/DE/Medientypen/Infomaterial/Broschüre-Das-BGW-Rückenkolleg.pdf?__blob=publicationFile **Das BGW-Rückenkolleg.**.

[B12] Ilmarinen J Tuomi K Eskelinen L Nygard CH Huuhtanen P Klockars M Summary and recommendations of a project involving cross-sectional and follow-up studies on the aging worker in Finnish municipal occupations (1981–1985) Scand J Work Environ Health 1991 17 Suppl 1 135 141 1792527

[B13] Hosmer DW Lemeshow S Applied logistic regression 2000 Wiley & Sons, New York

[B14] Kromark K Rojahn K Nienhaus A Bandscheibenbedingte Erkrankungen der Lendenwirbelsäule bei Krankenschwestern Trauma Berufskrankheit 2005 7 67 72 10.1007/s10039-004-0995-1

[B15] Arnstein P Caudill M Mandle CL Norris A Beasley R Self efficacy as a mediator of the relationship between pain intensity, disability and depression in chronic pain patients Pain 1999 80 483 491 10.1016/S0304-3959(98)00220-6 10342410

[B16] Smedley J Egger P Cooper C Coggon D Prospective cohort study of predictors of incident low back pain in nurses BMJ 1997 314 1225 1228 10.1136/bmj.314.7089.1225 9154024 PMC2126588

[B17] Yip YB A study of work stress, patient handling activities and the risk of low back pain among nurses in Hong Kong J Adv Nurs 2001 36 794 804 10.1046/j.1365-2648.2001.02037.x 11903709

[B18] Foster L Whitaker S Manual handling training and changes in work practices Occup Health (Lond) 1996 48 402 406 9283473

[B19] Lagerström M Hagberg M Evaluation of a 3 year education and training program. for nursing personnel at a Swedish hospital AAOHN J 1997 45 83 92 9146108

[B20] Freitag S Fincke-Junod I Seddouki R Dulon M Hermanns I Kersten JF Larsson TJ Nienhaus A Frequent bending - an underestimated burden in nursing professions Ann Occup Hyg 2012 56 697 707 10.1093/annhyg/mes002 22356807

[B21] Kromark K Metzing S Bartholomeyczik S Liersch A Nienhaus A Hilfsmittelausstattung und -nutzung in der stationären Altenpflege [Equipment and Use of Equipment in Nursing Homes] Gesundheitswesen 2006 68 41 47 10.1055/s-2005-858892 16463244

[B22] Simon M Tackenberg P Nienhaus A Estryn-Behar M Conway MP Hasselhorn HM Back or neck-pain-related disability of nursing staff in hospitals, nursing homes and home care in seven countries-results from the European NEXT-Study Int J Nurs Stud 2008 45 24 34 10.1016/j.ijnurstu.2006.11.003 17217951

[B23] LaFleur J Nelson RE Sauer BC Nebeker JR Overestimation of the effects of adherence on outcomes: a case study in healthy user bias and hypertension Heart 2011 97 1862 1869 10.1136/hrt.2011.223289 21586421

[B24] Faber A Giver H Stroyer J Hannerz H Are low back pain and low physical capacity risk indicators for dropout among recently qualified eldercare workers? a follow-up study Scand J Public Health 2010 38 810 816 10.1177/1403494810379891 20709891

[B25] Harling M Der Bedarf an Prävention und Gesundheitsförderungsmaßnahmen bei Beschäftigten in Pflegeberufen 2014 Edition Gesundheit und Arbeit, Hamburg

[B26] Hackmann T Nienhaus A Entwicklung der professionellen Pflege vor dem Hintergrund des demografischen Wandels Gefährdungsprofile-Unfälle und arbeitsbedingte Erkrankungen im Gesundheitsdienst und Wohlfahrtspflege 2010 2 ecomed MEDIZIN, Landsberg/Lech 96 112

[B27] http://nile.lub.lu.se/arbarch/saltsa/2003/wlr2003_07.pdf Hasselhorn H-M, Tackenberg P, Müller BH: **Working conditions and intent to leave the profession among nursing staff in Europe.**.

